# Chronic obstructive pulmonary disease and glucose metabolism: a bitter sweet symphony

**DOI:** 10.1186/1475-2840-11-132

**Published:** 2012-10-27

**Authors:** Aibek E Mirrakhimov

**Affiliations:** 1 Kyrgyz State Medical Academy named by I.K. Akhunbaev, Akhunbaev street 92, Bishkek 720020, Kyrgyzstan

**Keywords:** COPD, Dysglycemia, Insulin resistance, Obesity, Metabolic syndrome, Diabetes mellitus endothelial dysfunction, Vasculopathy

## Abstract

Chronic obstructive pulmonary disease, metabolic syndrome and diabetes mellitus are common and underdiagnosed medical conditions. It was predicted that chronic obstructive pulmonary disease will be the third leading cause of death worldwide by 2020. The healthcare burden of this disease is even greater if we consider the significant impact of chronic obstructive pulmonary disease on the cardiovascular morbidity and mortality.

Chronic obstructive pulmonary disease may be considered as a novel risk factor for new onset type 2 diabetes mellitus via multiple pathophysiological alterations such as: inflammation and oxidative stress, insulin resistance, weight gain and alterations in metabolism of adipokines.

On the other hand, diabetes may act as an independent factor, negatively affecting pulmonary structure and function. Diabetes is associated with an increased risk of pulmonary infections, disease exacerbations and worsened COPD outcomes. On the top of that, coexistent OSA may increase the risk for type 2 DM in some individuals.

The current scientific data necessitate a greater outlook on chronic obstructive pulmonary disease and chronic obstructive pulmonary disease may be viewed as a risk factor for the new onset type 2 diabetes mellitus. Conversely, both types of diabetes mellitus should be viewed as strong contributing factors for the development of obstructive lung disease. Such approach can potentially improve the outcomes and medical control for both conditions, and, thus, decrease the healthcare burden of these major medical problems.

## Introduction

Chronic obstructive pulmonary disease (COPD) defines a group of chronic inflammatory pulmonary disorders characterized by partially reversible airflow limitation [1]. The notion that COPD is primarily a lung disease has been challenged, and the broader definition of COPD as a systemic inflammatory syndrome has been proposed [2]. Evidence for this approach has been provided by strong associations with increased rates of cardiovascular diseases [3], anemia [4], musculoskeletal disease [5,6] and malignant conditions seen in COPD patients [7,8].

Moreover, it is predicted that COPD will be the third leading cause of death worldwide by 2020, which will impose an even greater burden on the healthcare system globally [9]. If the mortality from comorbid conditions associated with COPD is taken into count, then this disease poses an even greater impact on health outcomes. The common risk factors for COPD are air pollution, aging, respiratory infections, bronchial asthma (BA), low socioeconomic status [1].The COPD grading is presented in Table 1.

**Table 1:** **Global Initiative for Chronic Obstructive Lung Disease (GOLD) Grading system for COPD (Adapted from reference #) [**[1]**]**

**Grade**	**Severity**	**Spirometry findings**
**GOLD 1**	**Mild**	**FEV**_**1**_**/FVC<0.7 and FEV**_**1**_**≥80% predicted**
**GOLD 2**	**Moderate**	**FEV**_**1**_**/FVC<0.7 and 50%≤FEV**_**1**_**≥80% predicted**
**GOLD 3**	**Severe**	**FEV**_**1**_**/FVC<0.7 and 30%≤FEV**_**1**_**≥50% predicted**
**GOLD 4**	**Very Severe**	**FEV**_**1**_**/FVC<0.7 and FEV**_**1**_**>30% predicted**

**Abbreviations: *GOLD:*** Global Initiative for Chronic Obstructive Lung Disease**; *FEV***_***1***_***:*** Forced Expiratory Flow in 1 second**; *FVC:*** Forced Vital Capacity.

Type 2 diabetes mellitus (DM) and metabolic syndrome (MetS) are particularly common medical disorders and are leading causes of morbidity and mortality worldwide. Disturbances in glucose metabolism are more common in COPD patients than in COPD free individuals. COPD, MetS and type 2 DM are associated with advanced age, which may in part explain this observation. It is well known that offspring of affected parents are more likely to develop both COPD and type 2 DM. Smoking during pregnancy can in part explain the association between COPD and type 2 DM, due to delivery of low birth weight infants, which is a known risk factor for both diseases [10]. In addition, smoking can contribute to the occurrence of these diseases via its effects on systemic inflammation and oxidative stress [11,12]. However, the pathogenesis of glucose metabolism dysregulation is likely to be much more complex, whereby myriads of pathways are likely to be implicated, and much is still to be discovered and clarified.

On the other hand, type 1 DM and other forms of DM are much less common than type 2 DM [13], in part due to the growing burden of obesity and its associations with type 2 DM [14]. The risk factors for type 1 DM are not well understood and are of minor relevance to this manuscript. The major risk factors for type 2 DM are obesity, sedentary lifestyle, family history/genetic predisposition, aging, MetS, gestational diabetes and smoking.

Epidemiological data suggest that certain comorbid diseases, including DM are much more common in patients with COPD than in controls [3,15], and are associated with a worse COPD outcome [16]. As shown by Cazzola et al., patients with COPD have a higher burden of type 2 DM [17]. Sode et al. showed that patients with COPD have a greater burden of not only DM, but also of myocardial infarction, lung cancer, depression and hip fracture [18]. In contrast to these findings, Korean researchers did not find any association between COPD and greater DM prevalence, which may be related to high percentage of underweight subjects in the studied population [19].

The goal of this article is to summarize the published data on COPD and dysglycemic states. Firstly, the background of the problem and possible pathophysiological mechanisms will be discussed. Secondly, the article will focus on the clinical data and epidemiology of type 2 DM in COPD and the impact of both conditions on the clinical course of each other. Thirdly, we will briefly discuss the data on overlap syndrome, coexistence of obstructive sleep apnea (OSA) and COPD on the risk of type 2 DM.

### Pathophysiology of glucose metabolism disturbances in COPD

#### Weight as a risk factor for COPD

Obesity is a growing health concern worldwide, primarily due to modern lifestyle changes and sedentarism. Obesity is one of the major criteria of MetS and a well established risk factor for new onset type 2 DM [20].

Obesity is known to affect pulmonary function and lung volumes. Obesity is associated with a decrease in expiratory reserve volume (ERV) and functional residual capacity (FRC), due to its extrapulmonary restrictive component [21]. Aside from this, obesity can perpetuate both systemic and pulmonary inflammation, since excessive adipose tissue is able to produce various proinflammatory cytokines including interleukin-6 (IL-6) and tumor necrosis factor alpha (TNF-α). On the other hand, excessive central adiposity is associated with a decrease in adiponectin levels, which is known for its anti-inflammatory properties, and this can contribute to the pulmonary and vascular damage [22].

Leone et al. studied the association between the components of MetS and airflow obstruction in 129,965 men [23]. MetS was associated with low forced expiratory volume in one second (FEV_1_) (odds ratio (OR) 1.28; 95% confidence interval (CI) 1.20-1.37) and forced vital capacity (FVC) (OR 1.41; 95% CI 1.31-1.51), independently from possible confounding factors. Abdominal obesity was strongly associated with low FEV_1_ (OR 1.94; 95% CI 1.80-2.09) and FVC (OR 2.11; 95% CI 1.95-2.29). It is particularly pertinent to note that hyperglycemia, high blood pressure and dyslipidemia were related to airflow obstruction. This finding indirectly supports a potential impact of MetS and type 2 DM on the pathogenesis and clinical course of COPD.

Similar results were obtained in a study by Lam et al., who enrolled 7,358 adults [24]. These researchers showed that abdominal obesity was associated with airflow obstruction independently from smoking (OR 1.43; 95% CI 1.09-1.88). However, a study performed by Paek et al., who enrolled 4,001 individuals failed to document any relationship between MetS and airflow limitation [25].

Marquis et al. enrolled 38 subjects with COPD and 34 controls to test whether MetS and its components were more common in COPD patients [26]. Overall 47% of COPD patients had three or more MetS components in comparison with 21% of control subjects, and the prevalence of obesity was approximately two times higher in the COPD group.

Steuten et al. recruited 317 subjects with COPD to study the prevalence of weight distribution in COPD patients in a Dutch primary care setting [27]. Obesity was much more prevalent in mild to moderate COPD (16-24%) disease than in severe disease (6%) in this study.

A research group from the University of California in San Francisco enrolled 355 patients with COPD to study the impact of body composition on functional status in patients with various stages of COPD [28]. Obesity (BMI≥30 kg/m²) was diagnosed among 54% of study participants, which is much higher than in the general US population.

Guerra et al. in a nested case control study tested whether BMI was associated with emphysema and bronchitis phenotypes of COPD [29]. In subjects with bronchitis, 25% of COPD cases had a BMI≥28kg/m² compared with 16% of controls. Moreover, a BMI≥ 28kg/m² was associated with an increased risk of being diagnosed with bronchitis (OR 1.80; 95% CI 1.32-2.46), whereas, the presence of emphysema was related to decreased weight.

However, some previously published data suggests that low BMI is a risk factor for COPD [30] and others have reported that obese men have a lower annual FEV_1_ decline [31].

It is well known, that a low BMI in patients with COPD is considered to be a risk factor for all cause related and pulmonary related mortality [32]. This was first demonstrated by Celli et al., who validated a globally used prognostic tool, more known as the BODE Index. Others have validated three item questionnaire for a poor COPD related quality of life, with a low BMI being one of the criteria [33]. However, it is worth emphasizing that a decrease in BMI seen in advanced COPD is likely to be explained by a loss of fat free mass, such as skeletal muscle [34]. Skeletal muscle loss in advanced COPD may further contribute to the deterioration in the clinical course of the disease, which is pathophysiologically similar to cancer cachexia.

Landbo et al. recruited 1,218 men and 914 women with FEV_1_/FVC<0.7 and prospectively studied the impact of nutritional status on mortality in COPD from all causes [35]. Importantly, smoking status, spirometry data, BMI and respiratory symptoms were assessed at baseline and after 17 years. After adjustment for possible confounders, a lower BMI was associated with greater mortality compared with a normal or high BMI (relative risk (RR) 1.64; 95% CI 1.20-2.23); this association was strongest in patients with COPD.

The same research group tested whether a change in BMI was associated with mortality in a cohort of patients with COPD and controls [36]. Interestingly, a loss of > three BMI units was associated with increased all-cause mortality in controls (RR 1.63; 95% CI 1.38-1.92) as well in COPD patients (RR 1.71; 95% CI 1.32-2.23). Weight loss was related to an increased risk of COPD related mortality (RR 2.14; 95% CI 1.18-3.89), whereas weight gain was associated with increased mortality only in controls.

Jee et al. studied 1,213,829 Koreans to prospectively study the impact of weight on mortality in patients with COPD [37]. These researchers found that a lower BMI was associated with increased mortality from respiratory causes, whereas a relationship was observed between high BMI and increased mortality from cardiovascular diseases or malignancies.

These data highlight the unresolved issue of the “obesity paradox” seen in many chronic diseases, such as COPD and heart failure (HF), where a higher BMI is associated with a decrease in overall mortality. The discrepancies among COPD studies are likely because of different phenotypes of disease being enrolled, such as emphysema predominant and bronchitis predominant. Indeed, emphysema predominant phenotype is associated with a loss of skeletal muscle, rather than a loss of body fat, which may explain differences in mortality in patients with normal and low BMI. Thus, future studies are needed to address the fat free mass rather than BMI in studying the impact body weight on morbidity and mortality in patients with COPD. Indeed, loss of a skeletal muscle may explain a greater pulmonary function decline in patients with low BMI compared to patients with normal BMI as was shown by Watson et al. [31].

#### Adipokines and COPD

Adipose tissue is an active endocrine organ producing various substances, which regulate biological processes including glucose and insulin metabolism. Leptin and adiponectin are the most studied adipokines to date. Leptin may decrease the expression of insulin as well as decrease glucose uptake by pancreatic β cells. Adiponectin, on the other hand, may upregulate insulin secretion when it is physiologically required. The reader is referred to some comprehensive review articles on the topic of adipokines and glucose metabolism [38,39]. A summary of the adipokines and their relationship to DM and COPD is presented in Table 2.

**Table 2:** **Key proinflammatory cytokines and adipokines and their potential role in COPD and DM (Adapted from references #) [**[38-77]**]**

**Name**	**Major origin**	**Physiological function**	**Data on COPD**	**Data on DM**
CRP	Liver/Hepatocyte	Proinflammatory.	COPD is independently associated with increased levels of CRP. Moreover, CRP may predict the future onset of COPD.	Elevated CRP levels may predict the development of onset of type 2 DM.
TNF-α	Macrophages, other leukocytes and adipocytes	Proinflammatory and proapoptotic. Possible insulin antagonism.	COPD is independently associated with increased levels of TNF-α	May be a risk factor for the development of new onset type 2 DM.
IL-1	Macrophages, other leukocytes, dendritic cells etc.	Proinflammatory. Lymphocyte activation	IL-1 is implicated in the pathogenesis of COPD related inflammation.	An increase in IL-1β may predict the development of new onset type 2 DM.
IL-6	Lvier/Hepatocyte, macrophages, other leukocytes, adipocytes etc.	Proinflammatory. Upregulation of the synthesis of CRP and other proinflammatory cytokines in the liver.	COPD is independently associated with increased levels of IL-6.	IL-6 was shown to increase the risk for the new onset type 2 DM.
Fibrinogen	Liver/Hepatocyte	Proinflammatory. Active participation in coagulation.	COPD is independently associated with increased fibrinogen levels.	No data on fibrinogen and the risk of new onset type 2 DM.
Leptin	Adipocyte	Appetite regulation. Possible proinflammatory actions.	Leptin levels are increased in patients with COPD. May contribute to COPD related weight loss and pulmonary function decline.	Leptin may increase the risk of type 2 DM. Leptin may participate in the development of DM related complications via its proinflammatory actions.
Adiponectin	Adipocyte	Antiinflammatory. Increase in insulin synthesis and increase in insulin sensitivity.	Adiponectin levels are increased in patients with COPD and low BMI, which may explain decreased mortality from cardiovascular causes in advanced COPD.	Adiponectin may prevent the development of type 2 DM via its anti-inflammatory and proinsulin actions.
Resistin	Leukocytes	Proinflammatory and insulin antagonizing actions.	Resistin levels may be increased in COPD and mediate IR.	Resistin may directly participate in the development of IR.

**Abbreviations: *CRP:*** C-Reactive Protein**; *DM:*** Diabetes Mellitus**; *IL:*** Interleukin**; *TNF-α:*** Tumor Necrosis Factor alpha.

Overall there is a paucity of published data on COPD and adipokines. Some of the published reports suffer from flaws in assessing the effects of adipokines on lung function and on the course of COPD. Nevertheless, the current data on this topic will be briefly reviewed.

Broekhuizen et al. enrolled 14 patients with moderate COPD to study the relationship between sputum leptin and other inflammatory markers [40]. Sputum leptin levels were related to sputum C-reactive protein (CRP) (p<0.001) and TNF-α (p< 0.01).

Bruno et al. recruited 15 smokers without COPD and 27 smokers with mild to moderate COPD to study the pulmonary expression of leptin and its receptor [41]. They found that patients with COPD had increased pulmonary leptin expression, which is associated with airflow inflammation and obstruction.

A research group from Maastricht University, in the Netherlands enrolled 15 male subjects with bronchitis predominant COPD and 27 male subjects with emphysema predominant disease to study the association between systemic inflammation, leptin and energy balance in COPD [42]. Emphysematous patients had lower leptin concentration, most likely due to lower body weight (p= 0.02). A positive correlation between serum leptin and soluble TNF-α receptor levels was observed in patients with emphysema.

Opposing results were obtained by Takabatake et al., who recruited 31 people with COPD and 15 controls [43]. The mean BMI in COPD patients was significantly lower than in controls (BMI = 18.1 +/− 2.7 kg/m² versus 22.8 +/− 2.2 kg/m² respectively; p< 0.0001), which may explain the lower leptin levels seen in COPD patients (p < 0.05). These investigators failed to find any association between leptin levels and inflammatory biomarkers.

Karakas et al. enrolled 30 subjects with COPD and 20 healthy controls to assess the possible association between serum leptin levels and body composition [44]. Patients were grouped based on their BMI and serum leptin concentration. These researchers found an inverse relationship between serum leptin levels and BMI.

Wang et al. enrolled 57 patients with stable COPD and 31 controls to study the association between nutritional status and both leptin and resistin levels [45]. Leptin levels were positively correlated with resistin levels and both were associated with lower BMI. In support of this notion, a study performed by Al Mutairi et al., who studied the association between circulating resistin and inflammation showed a direct association between resistin levels and inflammatory obstructive airway disease [46]. These researchers speculated that resistin may be a novel disease marker and play a role in the development of insulin resistance (IR) in COPD.

Hansel et al. enrolled 429 subjects to study the relationship between leptin receptors and the rate of pulmonary function decline in patients with COPD [47]. They identified 21 single nucleotide polymorphisms (SNP) of the leptin receptor gene which were significantly associated with accelerated loss of lung function. This study provided information that certain leptin receptor gene polymorphisms may act as predisposing factors for COPD incidence among smokers, which could explain why not all smokers develop obstructive airway disease.

Calikoglu et al. recruited 26 patients with stable COPD, 16 patients with COPD exacerbation and 15 controls to study the association between leptin, TNF-α and nutritional parameters [48]. These investigators found that leptin levels were lower in patients with stable COPD in comparison with controls; however, TNF-α was higher in stable COPD than in controls and COPD exacerbation was associated with an increase in serum leptin and TNF-α. Dutch researchers found similar results with higher leptin levels during COPD exacerbation. It was speculated that this phenomenon could be explained by systemic inflammation and/or through high dose systemic corticosteroid treatment [49]. It was proposed that leptin may participate in the pathogenesis of pulmonary cachexia through its anorexigenic and metabolic effects [42].

Pan et al. enrolled 56 patients experiencing COPD exacerbation to study the association between dysglycemia, IR and in hospital clinical course [50]. The authors concluded that leptin induced IR and hyperglycemia prolonged hospital stay and adversely affected pulmonary function. Kythreotis et al. found that leptin levels were elevated during exacerbation and 2 weeks after the discharge [51].

However, an association between leptin and TNF-α was not found in a study performed by Yang et al. [52]. Small sample size and the population studied may underlie the lack of association in this work compared to other studies.

Tomoda et al. recruited 31 patients with COPD and 12 controls to study the role of adiponectin in patients with COPD [53]. Plasma adiponectin was higher among patients with COPD and correlated with residual volume (RV) (p<0.05), TNF-α (p<0.05) and was inversely correlated with BMI (p<0.01). These researchers speculated that high adiponectin levels may explain why COPD patients with a low BMI have decreased mortality due to cardiovascular causes.

Kirdar et al. enrolled 36 patients with COPD exacerbation and 17 controls to assess the role of adiponectin as a potential COPD biomarker [54]. Serum levels of adiponectin were higher among COPD patients (p<0.001); however, leptin metabolism was not altered in COPD exacerbation.

Miller et al. reported that patients with emphysema had a higher expression of pulmonary adiponectin [55], whereas Summer et al. showed that mice lacking adiponectin were prone to emphysema development [56].

Yoon et al. measured adiponectin levels in patients enrolled in the Lung Health Study to investigate the relationship between this adipokine and COPD [57]. These researchers found that increased adiponectin was related to a decrease in cardiovascular mortality, but was associated with an increase in mortality due to respiratory causes.

Breyer et al. studied the various adipokines and their expression in patients with COPD [58]. These researchers showed that patients with COPD had higher levels of CRP, IL-6, fibrinogen and adiponectin. Gender specific analysis demonstrated, that males with COPD had higher CRP, IL6 and fibrinogen levels, whereas, females with COPD had higher levels of CRP and fibrinogen only. Moreover, in female patients with COPD, leptin was correlated with CRP and fibrinogen, but this correlation was not observed in males with COPD. A negative correlation was shown between adiponectin and CRP in patients with COPD.

Based on the current data, leptin may propagate both pulmonary and systemic inflammation and together with resisitin contribute to the pathogenesis of related dysglycemia. Data on the adiponectin and mortality is quite intriguing and conflicting, which merits further research to explore potential mechanisms. In a mouse model of emphysema, it was shown that adiponectin may protect against the emphysema development. Thus, more studies are needed on the role of adipokines in the development and course of COPD, and whether they are not simply the markers of COPD progression.

#### COPD, systemic inflammation and oxidative stress

**Figure 1:**
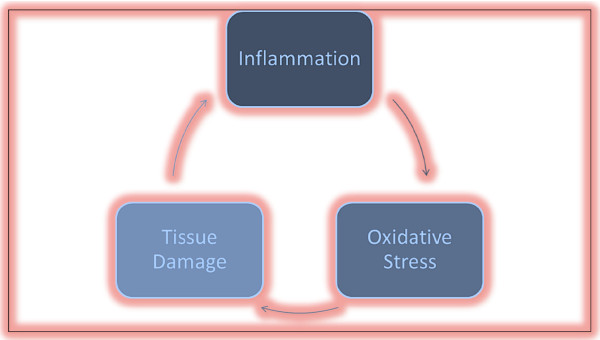
A simplified interrelationship between inflammation and oxidative stress.

As in many other chronic medical conditions, COPD is associated with low grade systemic inflammation. Various factors can contribute to this finding such as the spill of inflammatory mediators from the pulmonary system into the circulation, hypoxia, effects of obesity and hormonal disturbances. Current evidence suggests that excessive oxidative stress can be a risk factor for new onset type 2 DM and conversely, oxidative stress may be a consequence of new onset type 2 DM [59]. COPD as well as other pathologies, in which hypoxia is a feature is associated with an excessive oxidative state [60]. The reader is referred to a comprehensive review of hypoxia and metabolism for a more detailed discussion of this topic [61]. A summary of some of the key proinflammatory mediators and their relationship with DM and COPD is presented in Table 2. A simplified interrelationship between inflammation and oxidative stress is presented in Figure 1.

It is believed that inflammation increases the risk of future type 2 DM. Pradhan et al. studied 27,628 females to assess the impact of inflammatory biomarkers on the new onset type 2 DM [62]. They group found that the highest quartile of IL-6 and CRP gave a RR of 2.3 (95% CI 0.9-5.6) and 4.4 (95% CI 1.5-12.0) respectively. Spranger et al., studied 27,548 individuals to assess the relationship between inflammation and new onset type 2 DM [63]. These researchers found that interleukin-1beta (IL-1β) and IL-6 may be useful predictors of the development of new onset type 2 DM.

Landmark studies performed by Zinman et al. [64] and Hu et al. [65] showed a positive association between TNF-α and the development of IR and overt type 2 DM. Similar data came from the Atherosclerosis in Communities Study, which showed that low grade systemic inflammation predicts the incident type 2 DM [66].

Botelho et al. demonstrated that interleukin-1alpha (IL-1α) plays a major role in the development of neutrophilia in smoke induced inflammation in a mouse model [67].

Altaran et al. enrolled 50 patients with COPD to study the association between IL-6 and COPD severity [68]. These researchers showed an increase in IL-6 among patients with COPD and a possible independent contribution of IL-6 to airflow limitation. However, the results of the study cannot be generalized, since the majority of study participants had a history of sulfa mustard exposure. He et al. showed that a SNP in the IL-6 gene174G/C was associated with a rapid decline in FEV_1_ and COPD susceptibility among smokers [69]. Van Durme et al. showed that an increase in IL-6 was associated with an increased risk of COPD development; however, no association was found between polymorphisms in the IL-6 gene and such risk [70]. Data from further study, performed by Yanbaeva et al. supported the hypothesis of an increased risk of future COPD development associated with high IL-6, particularly for the IL-6 H2 haplotype [71]. Tkacova et al. studied the adipose tissue expression of CD40, a key receptor for the TNF in 20 patients with COPD [72]. These researchers showed that patients with severe COPD had a higher expression of CD40, which was inversely related to a lower partial pressure of oxygen. Thus, COPD related adipose tissue dysfunction may further enhance systemic inflammation and IR.

According to a meta-analysis performed by Gan et al., patients with COPD have higher levels of circulating neutrophils, CRP, fibrinogen and TNF-α. [73]. Vozarova et al., showed that a higher white blood cell count was associated with an increased risk of type 2 DM development [74]. Aside from this, elevated CRP has been shown to predict hospitalization and death in COPD [75], and CRP levels in COPD have a strong genetic background [76]. Moreover, a high CRP levels may act as a risk factor for future development of COPD among smokers [71,77]. The fact that only a small number of smokers develop COPD may be based on genetic alterations of common proinflammatory cytokines such as CRP and IL-6.

Indeed, the genetically mediated proinflammatory state in some individuals with COPD may underlie the clinical phenotype of patients with frequent COPD exacerbations. All of the findings may help in our understanding of the increase in cardiovascular morbidity and mortality, as well as the increased metabolic risk seen in COPD.

Both DM and COPD are associated with systemic inflammation and, on the other hand, systemic inflammation is associated with the disease risk, leading to a vicious cycle. Indeed, it was shown that certain genetic polymorphisms of common pro- inflammatory molecules may increase the risk for COPD in smokers.

#### Hypoxia and glucose metabolism

Pallayova et al. hypothesized that pancreatic β cells may be damaged by hypoxia, but the focus of their study was primarily on obstructive sleep apnea, which is associated with intermittent hypoxia, rather than chronic hypoxia seen in COPD [78]. However, both of these diseases may share some pathophysiological pathways, which may be mediated by the hypoxia inducible factor family (HIF) [61].

Based on the available data it is likely that different tissues have various metabolic responses to hypoxia. Interestingly, skeletal muscles under hypoxia have a higher glucose uptake [79], and increased sensitivity of skeletal muscles to insulin during exercise [80]. However, patients with COPD receiving oxygen therapy have less muscular IR [81]. This may be explained by the fact that long standing hypoxia may detrimentally affect skeletal muscle insulin sensitivity compared with acute muscular hypoxia seen in exercise.

HIF-1α expression is increased in hypoxic pancreatic β cell regions, undergoing programmed cell death, which may simply indicate an effect of hypoxia on the expression of HIF-1α, with the latter having no role in the pathogenesis of apoptosis. Alternatively HIF-1α may exert unique effects on the β cells [82]. Cheng et al. showed that HIF-1α has significant and beneficial effects on pancreatic β cell function, and hypothesized that HIF-1α may be a novel target to improve the function of insulin producing cells [83].

Important results have arisen from a study performed by Oltmanns et al., which exposed healthy volunteers to hypoxia under euglycemic clamp [84]. These researchers found that hypoxia caused glucose intolerance, and this phenomenon could be partly explained by an increase in epinephrine release.

The effects of hypoxia on the function of adipocytes seem to be straightforward. Chen et al. showed that hypoxia decreased the production of adiponectin and increased the production of plasminogen activator inhibitor-1 which may contribute to a greater cardiometabolic risk [85].

Aside from this, a hypoxia mediated increase in HIF-1α can induce adipose tissue fibrosis and resistance to insulin [86,87].

On the other hand, hyperglycemia is able to downregulate HIF-1α, and via this mechanism contributes to the pathophysiology of angiopathic conditions commonly seen in patients with DM [88].

Based on above findings, it is likely that hypoxia can mediate its detrimental effects on glucose metabolism and IR at least through the effects on insulin sensitivity at the level of adipose tissue and skeletal muscle.

#### Muscle metabolic pathways in COPD

IR at the level of skeletal muscle plays a role in the pathogenesis of type 2 DM. Earlier reports linked this phenomenon to a decreased amount of mitochondria in diabetic skeletal muscle, but new data suggest a decreased functional status of this cellular organelle [89]. From a theoretical point of view, it is plausible that COPD and associated hypoxia, can detrimentally affect mitochondria. However, it should be noted, that the evidence regarding the role of hypoxia in decreased glucose transport in skeletal muscle is controversial [90]. The information on this topic is relatively scant and will be briefly discussed below.

Wuyam et al. showed that patients with COPD had decreased aerobic capacity compared to healthy controls [91]. However, in contrast to their findings, others explained this association by a decreased level of physical activity at baseline, and a shift in type of muscle fiber, and, they, therefore, doubted the existence of an intrinsic mitochondrial problem in COPD [92].

Jakobsson et al. group reported that patients with advanced COPD had upregulated glycolysis in quadriceps femoris muscles, but lower muscular aerobic capacity [93]. This finding points toward the existence of an adaptation of skeletal muscle to hypoxia, by upregulating glucose carriers, such as GLUT-4.

COPD patients experience a shift in the skeletal muscle fiber [94], which may explain a decrease in oxidative capacity [91,95]; such a fiber shift has been shown to be associated with a greater COPD severity [96]. Patients with COPD have impaired β oxidation of fatty acids, electron transport chain and citric acid cycle, all of which are known to take place in mitochondria [97].

A key study performed by Green et al. showed that muscles from patients with COPD had lower levels of glucose transporter-4 (GLUT-4) compared with controls, which is known to be closely linked to insulin action [98]. However, as mentioned previously, others have failed to show any detrimental effects of hypoxia on glucose uptake by skeletal muscle cells.

Moreover, IR may synergistically impair muscle function, since it is associated with decreased muscle strength, even in patients without overt DM [99].

It is theoretically plausible, that longstanding hypoxia have different molecular effects on skeletal muscle metabolism compared with muscle hypoxia induced by physical exercise in subjects without pulmonary disease. However, this conclusion is not based on firm evidence and further research is needed to clarify the effects of hypoxia and COPD on glucose metabolism in skeletal muscle.

#### COPD and hormonal dysregulation

From a theoretical viewpoint the alterations in insulin opposing hormones can explain the association between the development of COPD and dysglycemia. However, it should be noted that the evidence is scant and controversial. Nevertheless, we will briefly discuss the data on COPD and hormones, which are associated with MetS and type 2 DM.

There are some reports linking abnormalities in androgen metabolism with the development of type 2 DM [100]. The exact mechanisms of these associations are not clear, but they may be related to the beneficial effects of testosterone on weight, insulin sensitivity and modulation of inflammation. Svartberg et al. showed that men with lower levels of free and total testosterone had lower numbers of FEV_1_ and FVC, independently from potential confounders [101].

Laghi et al. enrolled 101 patients with COPD and measured both free and total testosterone [102]. These researchers found that approximately 40% of patients with COPD were hypogonadal; however, they did not find any association between testosterone levels and pulmonary function. Notably, the prevalence of hypogonadism in patients with COPD has been shown to be greater than in an age-matched population without this disease [103], and the lack of association between androgens and pulmonary function could be explained by the small sample size and lack of a control group in this study.

In contrast to the study performed by Laghi et al. [102], Van Vliet et al. showed that hypogonadism was associated with decreased physical endurance, muscle weakness and proinflammatory state among patients with COPD [104]. It is difficult to prove causality between low testosterone and COPD, since multiple mechanisms are likely to act simultaneously. However, it is possible that hypoxia may underlie the development of hypogonadism in patients with pulmonary disease [105].

Catecholamine hormones are known to be insulin antagonists and contribute to the occurrence of hyperglycemia [106]. Kanstrup et al. showed an association between prolonged hypoxia and the development of enhanced catecholamine output in eight healthy men [107]. In another study, Scalvini et al. showed that patients with COPD had higher catecholamine levels which were independently related to a decrease in FEV_1_[108]. Bratel et al. showed that patients with severe nocturnal hypoxemia treated with long term oxygen therapy experienced a reduction in nocturnal norepinephrine, suggesting a potential causal role of hypoxia in the overactivation of the sympathetic nervous system in patients with COPD [109].

Abnormalities in the renin angiotensin aldosterone system (RAAS) are implicated in the development and pathogenesis of cardiovascular diseases, MetS and type 2 DM [110]. Farber et al., in two published studies, showed that patients with COPD had higher levels of aldosterone and an increase in plasma renin activity [111,112]. Stewart et al. found that patients with COPD had increased levels of vasopressin and aldosterone, which could be explained by COPD, related autonomic neuropathy [113]. Alternatively, the RAAS activity in patients with COPD can be upregulated by increased sympathetic tone, hypoxia and underlying comorbid states such as cardiac and renal pathologies. The importance of RAAS overactivation was highlighted by Mancini et al., who showed that drugs targeting the RAAS may improve survival in patients with COPD [114].

Vitamin D is implicated into the pathogenesis of myriad of extraskeletal diseases including both major types of DM [115,116]. This relationship can be explained by the anti-inflammatory and antioxidant effects of vitamin D as well as through its modulation of the RAAS activity. Moreover, patients with COPD exhibit a trend towards lower levels of vitamin D [117]. However, it is difficult to conclude causality, since patients with COPD may have a lower level of activity and, thus, have less sun exposure.

Evidence for an association between COPD and thyroid function is scant and predominantly negative [118].

To summarize, COPD is associated with abnormalities in metabolism of androgen hormones, vitamin D, catecholamines and RAAS. However, more studies are needed to clarify the role of these hormonal systems in the pathogenesis of COPD related IR.

### Clinical data on COPD and the risk of DM/MetS

#### COPD, reduced lung function and the risk of new onset type 2 DM

##### Reduced lung function and the risk of type 2 DM

Lazarus et al. in a prospective observational study showed that baseline values of FVC, FEV_1_ and maximal mid-expiratory flow rate (MMEF) are negatively associated with IR even after adjustment for potential confounders [119].

Ford et al. showed that FEV_1_ and FVC values at baseline were inversely associated with the incidence of type 2 DM [120]. It is important to note that restrictive, but not obstructive lung disease was associated with the incidence of new onset type 2 DM. Similar findings were shown by Paek et al. and Wannamethee et al. [25,121], who demonstrated that restrictive lung diseases were strongly associated with new type 2 DM. These results were statistically significant even after adjustment for confounding factors such as age, gender and weight.

Researchers from the Johns Hopkins University studied 11,479 subjects free of type 2 DM and followed them for nine years [122]. They found that lower FVC was independently associated with new onset type 2 DM in both men and women, independently from potential confounders such as age, weight and race. Hsiao et al. showed that reduced baseline FVC and FEV_1_ were independently related to a greater risk of future development of MetS [123]. A shared pathophysiology may underlie this association or alternatively, reduced lung volumes may simply be the markers of lower physical endurance in patients at risk for the development of MetS.

Similar data have been reported by Engstrom et al., who showed that reduced pulmonary function was inversely associated with the development of type 2 DM [124]. In a later study, Engstrom et al. showed that low FVC was associated with an increased risk of IR and the development of new onset type 2 DM [125].

In a recent cross-sectional study, Kwon et al. showed that reduced FEV_1_ and FVC are directly related to the new onset type 2 DM [126]. The results stayed significant even after adjustment for age, weight, health literacy and exercise.

Cross-sectional studies performed in Japanese [127] and Korean men [128,129] showed an association between reduced FVC and an increased risk of both MetS and type 2 DM.

However, the association between type 2 DM and reduced lung function should be interpreted with caution. It is well known that most of the participants enrolled in these studies were obese, and obesity is associated with reduced respiratory muscle performance, which could underlie this relationship [130,131].

Heianza et al. observed 5,346 men without DM or pulmonary disease to study the impact of decreased lung function on the incidence of type 2 DM [132]. They showed that reduced pulmonary function was associated with increased incidence of type 2 DM independently from BMI, smoking status and baseline glycated hemoglobin (hazard ratio (HR) 13; 95% CI, 1.01-1.26).

Thus, the observed association could be explained by three possibilities: reduced lung performance and incidence of type 2 DM share similar pathophysiological pathways (such as low grade inflammation); secondly, that reduced lung performance could be just a marker of reduced physical endurance; thirdly, that reduced lung function may simply portray the overall health status of individuals prone to develop type 2 DM, with obesity being the most important confounding factor.

A summary of the key studies on the topic of reduced pulmonary function and a risk of type 2 DM is presented in Table 3.

**Table 3:** Key studies on pulmonary volumes and the risk of new onset type 2 DM

**First author and year**	**Country**	**Study design**	**Population studied**	**Findings**
Lazarus et al. [119]; 1998	USA	Prospective cohort study with a mean follow up of 20.9 years	n:1,050 men (with no self-reported DM) included in the final analysis mean age: 41.4 years mean BMI: 25.6 kg/m²	Reduced FVC, FEV_1_ and MMEF were associated with greater fasting insulin and fasting insulin resistance after logistic regression analysis.
Engström et al. [124]; 2002	Sweden	Prospective cohort study with a mean follow up of 13 years	n: 382 non-diabetic men age at the beginning of the study:55 years mean BMI: 24.4-24.7 years (depends on the pulmonary VC subgroup)	15 new cases of type 2 DM were diagnosed during the follow up. DM and log glucose were inversely associated with baseline VC.
Engström et al. [125]; 2003	Sweden	Population based cohort study with a mean follow up of 13.9 years for men and 9.4 years for women	n: 2,332 non-diabetic subjects (men-1,436, 61.57%) mean age for men: 44.0-44.9 years (depends on the pulmonary FVC subgroup) mean age for women: 49.4-50.4 years (depends on the pulmonary FVC subgroup) mean BMI for men: 24.2-24.9 kg/m²(depends on the pulmonary FVC subgroup) mean BMI for women: 23.2-23.9 kg/m²(depends on the pulmonary FVC subgroup)	The association between baseline FVC and IR at follow up remained statistically significant after adjustment for gender, age at screening, follow up time, smoking/tobacco consumption, BMI and waist to hip ratio, physical activity and baseline log glucose
Kwon et al. [126]; 2012	Korea	Prospective cohort study with a follow up of 5 years	n: 9,220 men non-diabetic at baseline mean age: 41.4 years mean baseline BMI: 24.4 kg/m² for patients without type 2 DM at follow up and 26.7 kg/m² for patients with type 2 DM at follow up	207 patients developed type 2 DM with the incidence of 2.2%.
				FEV1 and FVC were negatively associated with type 2 DM.
				In patients with BMI<25 kg/m2 the lowest quartile of FVC and FEV1 had OR of 2.15 (95% CI 1.02-4.57) and 2.19 (95% CI 1.09-4.42) for incident type 2 DM.
Heianza et al. [132]; 2012	Japan	Observational study with a 4 year follow up	n: 5,346 (100% men) mean age: 48.6 years mean BMI: 23.3 kg/m²	Compared with the highest FEV1 quartile, lower quartiles were associated with 1.79-1.86 greater risk for the new onset type 2 DM (95% CI, 1.10-2.91).

**Abbreviations: *BMI****:* Body Mass Index; ***CI****:* Confidence Interval; ***FEV***_***1***_: Forced Expiratory Volume in 1 second; ***FVC****:* Forced Vital Capacity; ***HR****:* Hazard Ratio; ***MMEF****:* Maximal Mid-Expiratory Flow; ***OR****:* Odds Ratio; ***VC****:* Vital Capacity.

##### COPD and the risk of type 2 DM

Several studies assessed the impact of COPD on the risk of new onset type 2 DM. Rana et al. analyzed data from the Nurses’ Health Study from 1988–1996 which enrolled 103,614 females [133]. Study participants were followed for eight years, and COPD was found to have a multivariate RR of 1.8 (95% CI 1.1-2.8) for new onset type 2 DM. It is vital to note that the diagnosis of BA was not associated with an increased risk of type 2 DM. However, these results should be interpreted with caution, since spirometry data were not available and the diagnosis of pulmonary disease was based on the patients’ questionnaires.

**Figure 2:**
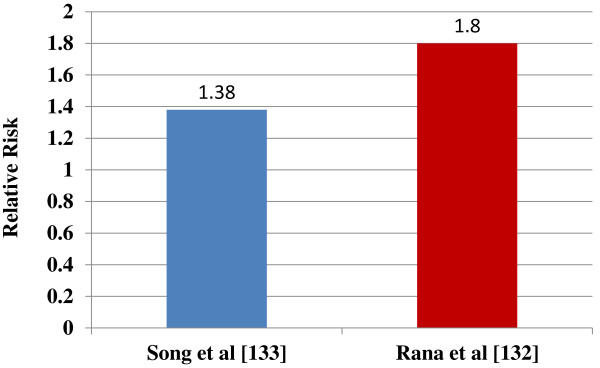
**COPD and the risk of type 2 DM (adapted from references) **[133,134]**.**

Song et al. analyzed data of the Women’s Health Study in which 38,570 women without DM were enrolled [134]. As in the previous study, the diagnosis of pulmonary disease was assessed by the presence of self-reported disease. The study participants were followed for a median of 12.2 years. Women who had reported physician diagnosed COPD had a multivariate RR of 1.38 (95% CI 1.14-1.67) for new onset type 2 DM. In contrast to the previous study, these investigators found that BA was associated with an increased risk of type 2 DM with a RR of 1.37 (95% CI 1.2-1.57). Adapted data from the references [132] and [133] are presented in Figure 2.

Feary et al. analyzed the primary care records of 1,204,100 individuals aged over 35 years from 311 primary care practices in the United Kingdom [3]. They showed an OR of 2.04 (95% CI 1.97-2.12) for the development of new onset type 2 DM associated with physician diagnosed COPD.

Bolton et al. recruited 56 non-hypoxemic COPD patients and 29 healthy controls to study the potential association between inflammation and IR [135]. Patients with COPD had higher insulin levels, which were related to inflammatory markers such as CRP, IL-6 and soluble receptors for TNF-α. Indeed, the COPD related inflammatory state may underlie the pathogenesis of an increased risk of type 2 DM in these patients.

In contrast, Bayliss et al. failed to observe any effect of COPD exacerbation on worsened long term glycemic control in patients with type 2 DM [136]. However, as was acknowledged by these researchers, the studies using community electronic records are subject to some limitations such as recall and selection bias; thus, the results should be interpreted with caution.

Based on the current evidence, COPD should be approached as a risk factor for the new onset type 2 DM. This risk is based on myriad of pathological pathways discussed in this manuscript. A summary of the key studies on COPD and a risk of type 2 DM is presented in Table 4.

**Table 4:** Key studies assessing the risk of IR/new onset type 2 DM and control of DM in patients with COPD

**First author and year**	**Country**	**Study design**	**Population studied**	**Findings**
Feary et al. [3]; 2010	UK	Cross-sectional study using computerized records from 311 primary care practices	n: 1,204,100 (Men-618,090 subjects-51.3%) age: 35–44 years-327,800 subjects (27.2%), 45–54 years-274,375 subjects (22.8%), 55–64 years-254,472 subjects (21.1%), 65–74 years-177,102 subjects (14.7%) and ≥75 years- 170,361 subjects (14.2%)	COPD had OR of 2.04 (95% CI 1.97-2.12) for the development of new onset type 2 DM.
Song et al. [134]; 2010	USA	Prospective cohort study with a median follow up of 12.2 years	n:38,570 non-diabetic women mean age-:57.8 years(for patients with physician diagnosed COPD) mean BMI: 26.1 kg/m²(for patients with physician diagnosed COPD)	COPD had a multivariate adjuster RR of 1.38 (95% CI 1.14–1.67) for a new onset type 2 DM.
			Data on BMI not provided	
Bolton et al. [135]; 2007	UK	Cross-sectional study	n: 56 patients (30 men) with non-hypoxemic COPD and 29 healthy subjects (15 men) mean age: 66.7 years for COPD patients and 62.9 years for healthy controls mean BMI: 25.7 kg/m² for COPD patients and 26.5 kg/m² for healthy subjects	Patients with COPD had greater levels of CRP, IL-6 and TNF-α sr I and sr II.
				IR assessed by HOMA was 1.68 in COPD patients vs. 1.13 in healthy subjects.
				Log_10_ IL-6 and BMI were found to be predictive for IR in stepwise regression analysis
Bayliss et al. [136]; 2011	USA	Prospective cohort study in patients with established type 2 DM	2,332 develop chronic pulmonary disease exacerbation (women-1,244, 53.34%), 582 (women-318, 54.64%) developed cancer and 2,959 developed depression (women-1,670, 56.44%) mean age at index date: 62.04 years for depression, 63.2 years for chronic pulmonary disease exacerbation and 65.53 years for cancer mean BMI: 31.09 kg/m² for cancer, 32.01 kg/m² for depression and 32.45 kg/m² for chronic pulmonary disease exacerbation	The researchers did not find any effect of chronic pulmonary disease exacerbation, depression and cancer on the glycated hemoglobin.

**Abbreviations**: ***BMI****:* Body Mass Index; ***CI****:* Confidence Interval; ***COPD****:* Chronic Obstructive Pulmonary Disease; ***CRP****:* C-Reactive Protein; ***DM:*** Diabetes Mellitus; ***HOMA****:* Homeostatic Model Assessment; ***IL-6****:* Interleukin-6; ***OR****:* Odds Ratio; ***RR****:* Relative Risk; **sr**: Soluble Receptor.

##### Treatment of COPD and the risk of hyperglycemia

Patients with COPD are often treated with corticosteroids, either inhaled (typically stable COPD) or systemic (typically COPD exacerbation) [1]. Corticosteroid therapy is associated with a decreased rate of FEV_1_ and pulmonary function decline and fewer disease exacerbations.

However, it is well known that systemic corticosteroid therapy is associated with multiple undesired effects, including the development of dysglycemia and overt DM [137]. Niewoehner et al. showed that systemic glucocorticosteroid therapy was significantly associated with the development of medically relevant hyperglycemia in 271 patients with COPD exacerbation [138]. Burt et al. showed that patients with COPD treated with systemic corticosteroids had a circadian rise of glucose predominantly in the afternoon and evening [139].

Despite being useful in the management of COPD exacerbation, long term systemic corticosteroid therapy is associated with the development of respiratory muscle damage with resultant decreases in both inspiratory and expiratory muscle strengths [140,141]. Detrimental effects of systemic corticosteroids on respiratory performance have been confirmed in patients with BA [142] and in patients with normal baseline pulmonary function [143].

In contrast to systemic corticosteroids, inhaled corticosteroids have much more favorable side effect profile, which is explained by the route of administration and a lower corticosteroid dose being administered [144]. A recently published pooled analysis of 34 studies using inhaled steroids in patients with BA or COPD failed to show any association with new onset type 2 DM or hyperglycemia [145]. However, higher doses of inhaled corticosteroids were associated with higher glucose levels in treated patients in one published study [146].

Thus, it is critically relevant to address the risk of new onset type 2 DM in patients with COPD being treated with high doses of inhaled corticosteroids due to a greater prevalence of other risk factors for DM in this population.

#### The impact of DM/Mets on the lung

#### Effects of DM on the pulmonary vasculature and diffusing capacity

DM is directly implicated in the development of vascular damage to both large and small arteries or macroangiopathy and microangiopathy respectively [147,148]. Therefore, it is plausible that long standing DM detrimentally affects the alveolar capillary bed. Key studies in this area will be briefly reviewed.

Guazzi et al. investigated the association between type 2 DM with or without HF and the worsening pulmonary diffusion capacity to carbon monoxide (DLCO) [149]. These researchers recruited 15 healthy controls and three groups 15 patients with type 2 DM, type 2 DM+HF and HF alone. They found that patients with both HF and type 2 DM had a lower level of DLCO, which is a known surrogate marker for the alveolar capillary membrane morphological and functional status. Subsequently, the researchers tested the effects of regular insulin on DLCO. They found that the administration of insulin improved DLCO in patients with type 2 DM and type 2 DM+HF, but not in patients with HF alone. The same group tested the effects of administered regular insulin on the alveolar gas conductance in 19 never smokers with type 2 DM [150]. They found an association between type 2 DM and decreased alveolar gas conductance. Administered insulin improved gas conductance, which was believed to occur through the decrease in alveolar capillary membrane impedance.

In fact, the observed beneficial effects of insulin on the pulmonary variables were attractive and further studies were carried out to explore an association with the use of inhaled insulin [151]. However, unfortunately, some studies pointed towards the potential adverse effects of inhaled insulin, in terms of the occurrence of a cough, and a potential reduction in DLCO and FEV_1_[152]. Some experts doubted the usefulness of inhaled insulin, due to an age associated decline in pulmonary function and reserve [153] and the fact that smoking can significantly alter the absorption of inhaled insulin [154]. However, in contrast a recent trial did not show any long term detrimental effects of inhaled insulin on pulmonary variables in patients with BA [155] and a second study did not find any harmful effects of inhaled insulin on pulmonary function in patients with type 2 DM [156]. Despite, these optimistic data, more research is needed before inhaled insulin can be recommended in diabetic patients with or without pulmonary disease.

Guvener et al. enrolled 25 patients with type 2 DM and 12 healthy controls to assess the impact of type 2 DM on alveolar capillary permeability, measured using the ratio of DLCO to alveolar ventilation (VA) [157]. The study demonstrated a statistically significant decrease in alveolar capillary capacity in patients with type 2 DM compared with healthy controls. Age, duration of type 2 DM and the presence of microalbuminuria were associated with the presence of alveolar capillary exchange abnormality. It is pertinent to note that microalbuminuria was predictive for the presence of pulmonary capillaropathy, and it is theoretically plausible that both diabetic related complications share a similar pathophysiology.

Researchers from the Southwestern University, USA enrolled 65 never smokers with type 2 DM to assess the status of pulmonary microvascular reserve [158]. Both non-obese (BMI<30 kg/m²) and obese (BMI≥30 kg/m²) patients with type 2 DM had reduced levels of the measured pulmonary function variables during exercise. However, the observed changes in obese patients were not fully explained by the baseline lung volumes. Moreover, the presence of retinopathy, neuropathy, microalbuminuria and control of diabetes were associated with a reduced pulmonary microvascular reserve.

It is important to mention the findings of Minette et al., who demonstrated that never smokers with type 1 DM had a thicker alveolar capillary membrane [159]. However, these researchers failed to find any association between type 1 DM and worsened alveolar gas conductance. Schernthaner et al. were unable to find any adverse impact of type 1 DM on lung function [160]. However, it should be noted, that the cross-sectional design of the above studies pose limitations on the study conclusion, since the study participants could be tested at the time, when disease was at a subclinical stage.

In contrast to these negative studies of type 1 DM and lung diffusing capacity, the researchers from Russia demonstrated that a long standing type 1 DM was associated with reduced lung volumes and decreased alveolar capillary gas exchange [161]. However, it is important to keep in mind that patients with type 1 DM are generally younger and may have less pulmonary damage resulting from clinically detectable pulmonary disease compared with type 2 DM at the time of the actual study, due to a greater lifetime exposure to noxious stimuli and an age related physiological decline in lung function.

Asanuma et al. compared FVC and DLCO in patients with DM without pulmonary disease to healthy age-matched controls [162]. They found that patients with DM have lower DLCO and FVC in comparison with controls. This reduction was significantly related to the duration of DM and the presence of diabetic retinopathy.

Sandler et al. showed that patients with type 1 DM had a lower pulmonary capillary flow with a resultant decrease in DCLO, which was associated with the duration of DM [163]. Cooper et al. showed that older patients (>35 years) with type 1 DM had lower DLCO compared with controls, which might be explained by an underlining pulmonary microvascular disease [164].

Ramirez et al. enrolled 18 patients with type 1 DM and assigned them either to a standard insulin regimen or to intensive treatment with measurement of glycated hemoglobin at quarterly intervals [165]. These researchers followed study participants for six years with measurement of DLCO and FVC. Participants from the standard treatment group had higher levels of glycated hemoglobin and worse values of DLCO and FVC. The researchers speculated that the treatment of DM itself may prevent the development of pulmonary dysfunction pointing toward a causative role. In a later study, the same group supported these findings, by demonstrating diminished DLCO and greater breathing effort in patients with type 1 DM [166].

Strojek et al. showed that patients with type 1 DM related complications had lower levels of DLCO compared with patients with type 1 DM and no complications [167]. Similar results were found by Innocenti et al., who showed an association between urinary albumin excretion and diminished DLCO [168]. Schnack et al. showed that patients with type 1 DM and microalbuminuria had lower values of FEV_1_, vital capacity (VC) and DLCO compared with patients without microalbuminuria and to controls [169].

Saler et al. compared DLCO between 80 healthy subjects 44 patients with type 1 DM and 68 patients with type 2 DM [170]. DLCO and the ratio of DLCO to VA were significantly decreased in patients with both types of DM, but not in the control group. As shown in the above mentioned studies the urinary albumin excretion was inversely correlated with a decline in pulmonary diffusing capacity. It is essential to note that even children with type 1 DM have lower DLCO, which can be viewed as an early marker of DM related microangiopathy [171].

Sinha et al. showed that patients with type 2 DM and microangiopathy had lower DLCO in comparison with patients with type 2 DM and no microangiopathy [172].

Boulbou et al. enrolled 16 patients with type 1 DM, 33 patients with type 2 DM and 22 healthy controls to study the expression of adhesion molecules in all groups [173]. Patients with both types of DM had lower levels of DLCO corrected for alveolar ventilation and this was inversely related to increased expression of E-Selectin. Indeed, based on the current data DM can be considered as a risk factor for pulmonary capillaropathy with subsequent alteration in DLCO.

In contrast, a research group from Israel failed to find any association between the presence of DM and decreased DLCO or other pulmonary function parameters [174]. However, some study limitations, such as a small sample size and a relatively young age of the subjects may explain these negative results.

Robust data indicate that DM leads to pulmonary microangiopathy through the mechanisms similar to the DM related nephropathy. This is based on the fact the presence of DM related renal disease was significantly associated with the presence of pulmonary capillary dysfunction. A summary of the key studies on the impact of DM on pulmonary diffusing capacity is presented in Table 5.

**Table 5:** Some of the key studies assessing the impact of type 2 DM/MetS on the pulmonary function and COPD

**First author and year**	**Country**	**Study design**	**Population studied**	**Findings**
Guazzi et al. [149]; 2002	Italy	In hospital study assessing the effects of regular insulin on the alveolar-capillary conductance.	n: 19 patients (11 men) with type 2 DM and normal cardiac function. mean age: 59.9 years mean weight: 75.8 kg.	DLCO and alveolar capillary membrane conductance were increased by 12% and 14% respectively by insulin therapy.
		DLCO and its subcomponents were measured.		
Guazzi et al. [150]; 2002	Italy	Cross-sectional study.	n: 30 patients with HF (19 men), 15 patients with type 2 DM (8 men) and 15 controls (8 men). mean age: 61.1 years for controls, 62.3 years for patients with type 2 DM, 62.8 years for patients with type 2 DM and HF and 64.1 years for patients with HF alone. mean weight: 75.2 kg for patients with HF and type 2 DM, 76.1 kg for patients with type 2 DM, 76.7 kg for patients with HF and 77.8 kg for controls.	Patients with type 2 DM had a lower DLCO than controls.
				Regular insulin improved DLCO in patients with type 2 DM and type DM and HF, with no improvement in patients with HF alone. The improvement in patients with type 2 DM and HF was greater than in patients with type 2 DM alone.
Guvener et al. [157]; 2003	Turkey	Cross-sectional study.	n: 25 patients with type 2 DM (9 men) and 12 healthy controls (4 men). mean age: 56.3 years for patients with type 2 DM and 50.1 years for controlsmean BMI: 29.9 kg/m² for patients with type 2 DM and 29.5 kg/m² for controls.	Patients with type 2 DM had lower ratio of DLCO to VA. In a stepwise regression model with inclusion of age, duration of type 2 DM and microalbuminuria, only microalbuminuria was found to be independent predictor of DLCO/VA.
Chance et al. [158]; 2008	USA	Cross-sectional study assessing alveolar capillary bed in patients with type 2 DM.	n: 69 never smokers with type 2 DM and no overt cardiopulmonary disease (46% women) vs. 45 controls (45% women). mean age: 45 years for controls, 49 years for patients with type 2 DM and BMI>30 kg/m² and 45 years for patients with type 2 DM and BMI<30 kg/m². mean BMI: 28.8 kg/m2 for controls, 27.4 kg/m² and 34.4 kg/m² for aforementioned type 2 DM groups respectively.	Both non-obese (BMI<30 kg/m²) and obese (BMI≥30 kg/m²) patients with type 2 DM had reduced levels of the measured pulmonary function variables during exercise. However, the observed changes in obese patients were not fully explained by the baseline lung volumes. Moreover, the presence of retinopathy, neuropathy, microalbuminuria and control of diabetes were associated with the reduced pulmonary microvascular reserve
Niranjan et al. [166]; 1997	USA	Prospective observational study of cardio-pulmonary function at 7 years of follow up. Aerobic exercise capacity was measured with cycle ergometry. Lung volume and diffusing capacity were measured with rebreathing technique and ventialatory power was measured by esophageal balloon technique.	n: 18 subjects with type 1 DM (11 men) and 14 controls (10 men). mean age: 31 years for controls and 39 for patients with type 1 DM. mean BMI: 22 kg/m² for controls and 24.91-25.49 kg/m² among patients with type 1 DM.	Patients with poor glycemic control had worse restriction of lung volume, pulmonary diffusing capacity and membrane diffusing capacity. Cardiac stroke index was reduced among subjects with poor glycemic control.
				In the long-term analysis the rate of FEV_1_ and FVC decline was similar to those without DM.
Saler et al. [170]; 2009	Turkey	Cross-sectional study assessing alveolar capillary bed in patients with type 1 and type 2 DM.	n: 44 subjects with type 1 DM (29 women), 68 subjects with type 2 DM (49 women) and 80 controls (58 women). mean age: 32.52 years for patients with type 1 DM, 52.4 years for patients with type 2 DM and 40.08 years for controls. mean BMI: 24.4 kg/m² for patients with type 1 DM, 27.0 kg/m² for patients with type 2 DM and 25.6 kg/m² for controls.	DLCO and the ratio of DLCO to VA were significantly decreased in patients with both types of DM, but not in control group.
Wanke et al. [184]; 1991	Austria	Cross-sectional study.	n: 36 patients with type 1 DM (31 men) and 40 controls (33 men). mean age: 33 years for patients with DM and 27 years for controls. mean BMI: 24.3 kg/m² for patients with DM and 22.3 kg/m² for controls.	Patients with type 1 DM had significantly lower inspiratory VC, which was in part explained by reduced maximal sniff transesophageal and transdiaphragmatic pressures in patients with type 1 DM.
Walter et al. [194]; 2003	USA	Prospective observational study with a follow up>15 years using the data of the Framingham Heart Study.	n: 3,254 (1,547 men) with 280 subjects having type 2 DM mean age: 53.9 years for patients without type 2 DM and 59.6 years for patients with type 2 DM. mean BMI: 27.3 kg/m² for patients without type 2 DM and 30.67 kg/m² for patients with type 2 DM.	Patients with type 2 DM had a lower mean FEV_1_, FVC. The FVC/FEV_1_ ratio was slightly higher in patients with type 2 DM.
				Higher fasting glucose levels had association with decreased FEV_1_, FVC and FVC/FEV_1_ ratio (only in current smokers).
Lawlor et al. [195]; 2004	UK	Cross-sectional study.	N: 3,911 women (9.8% had type 2 DM).	FEV1 and FVC were inversely related to the HOMA score.
			Age: post-menopausal women aged 60–79 years	After adjustment for age, anthropometric variables, smoking, physical activity, childhood and adult social class and respiratory medications the higher FEV_1_ and FVC were associated with decrease in HOMA score of 3% and 5% respectively.
			Mean BMI: not provided.	Patients with higher values of FEV_1_ and FVC had lower prevalence of DM.
Davis et al. [196]; 2004	Australia	Prospective study using the data from the Fremantle Diabetes Study with a mean follow up of 7 years.	n: 125 subjects with type 2 DM. mean age: 61.5 years mean BMI: 29.9 kg/m²	Higher follow up fasting glucose, greater levels of follow up HbA1c and mean updated HbA1c were associated with a decrease in measured pulmonary parameters.
		FEV1, FVC, VC and PEF were measured at baseline and during follow up.		Decreased FEV_1_ was found to be independent predictor of all-cause mortality.
McKeever et al. [197]; 2005	UK	Study analyzing the data of the NHANES III.	n: 4,257 (1,943 men) mean age: 37 years mean BMI: 27.0 kg/m²	Patients with higher 2 hour 75 g glucose tolerance test had lower levels of FEV_1_ and FVC.
				Patients with a history of DM had lower levels of FEV_1_ and patients with poor control of DM had lower FEV_1_ than patients with controlled DM.
Litonjua et al. [198]; 2005	USA	A nested case–control study using the data of Normative Aging Study.	n: 352 men who developed type 2 DM and 352 controls (all men) mean age: 43.1 years for patients with type 2 DM and 43.2 years for controls. mean BMI: 26.81 kg/m² for patients with type 2 DM and 25.31 kg/m² for controls.	Patients with type 2 DM had lower FEV_1_ and FVC values (but not FEV_1_/FVC) many years prior to the diagnosis of type 2 DM.
				However, there was no difference in the rate of annual decline of FEV1 and FVC in patients with type 2 DM and controls.
Baker et al. [207]; 2006	UK	Cross-sectional study in patients admitted with acute exacerbation of COPD.	n: 284 subjects (167 men) mean age: from 72.9 to 76.7 years (depends on the glucose quartile, with higher glucose associated with older age) mean BMI: not provided	The RR of death and long hospital stay was greater in patients with glucose from 7.0-8.9 mmol/l and >9.0 mmol/l (RR 1.46 and 1.97 espectively), which was independent from age, gender, COPD severity and prior diagnosis of DM.
		The patients were divided into 3 groups based on the glucose quartile (<6.0mmol/l, 6.0-6.9 mmol/l 7.0-8.9 mmol/l and >9 mmol/l)		
				Each mole increase in glucose was associated with 15% greater risk for adverse clinical outcome.
				Higher glucose level was associated with the isolation of Staphylococcus Aureus from the sputum.
Chakrabarti et al. [208]; 2009	UK	An observation study on the effects of hyperglycemia on the outcome of non-invasive ventilation (NIV) during COPD exacerbation.	n: 88 in hospital patients (39 men). mean age: 70 years mean BMI: data not provided	Random blood glucose≥7.0 mmol/l was independently associated with an adverse NIV outcome such as NIV failure and greater risk for pneumonia.
Küpeli et al. [209]; 2010	Turkey	An observational study assessing the correlation between the presence of MetS and COPD exacerbation rate.	n: 106:29 patients with MetS (24 men) and 77 patients without MetS (67 men). mean age: 64.9 years for patients with MetS and 67.3 years for patients without MetS.mean BMI: 30.3 kg/m² for patients with MetS And 27.2 kg/m² for patients without MetS.	The mean COPD exacerbation rate was 2.4 in MetS group compared to 0.68 in the control group. Mean length of each exacerbation was 7.5 days in patients with MetS compared to 5 days in patients without MetS. Serum C-reactive protein, fasting blood glucose, and triglycerides were positively correlated with COPD exacerbation rate.

**Abbreviations: *BMI:*** Body Mass Index**; *COPD:*** Chronic Obstructive Pulmonary Disease**; *DLCO:*** Diffusion Capacity for Carbon Monoxide; ***DM****:* Diabetes Mellitus; ***FEV***_***1***_*:* Forced Expiratory Volume in 1 second; ***FVC****:* Forced Vital Capacity; ***HF****:* Heart Failure; ***HOMA****:* Homeostasis Model Assessment; ***NHANES****:* National Health and Nutrition Examination Survey; ***MetS****:* Metabolic Syndrome; ***NIV****:* Non-Invasive Ventilation; ***PEF****:* Peak Expiratory Flow; ***RR****:* Relative Risk; ***VA:*** Alveolar Ventilation; ***VC****:* Vital Capacity.

#### Effects of DM on the respiratory muscles and physical endurance

DM is associated with the development of muscle dysfunction, which may not be clinically detectable, and yet may contribute to the occurrence of diabetic complications [99,175]. Moreover, DM is independently associated with decreased physical performance and endurance [176]. This association can be explained by the impact of diabetic neuropathy and the, directly detrimental, effects of DM on the muscle.

The diaphragm, which is a major respiratory muscle, has the morphological structure of skeletal musculature and can be targeted by DM related hyperglycemia [177].

Fierro et al. showed that patients with DM had decreased phrenic nerve conduction velocity, which can underlie a diminished respiratory performance [178]. Several other reports have highlighted the detrimental effects of DM on the diaphragm, which are probably mediated via phrenic neuropathy [179-181].

Polotsky et al. showed in a mouse model that the diaphragm glycosylation is associated with decreased hypercapnic ventilator response [182]. In fact, the muscular proteins are prone to glycation, with resultant impaired functionality, which likely explains the pathophysiology of DM related diaphragm weakness [183]. Wanke et al. supported the notion of impaired respiratory muscle performance, by showing a decrease in inspiratory VC in patients with type 1 DM [184].

Tang et al. reported a case of patient with type 2 DM and associated phrenic neuropathy complicated by the development of acute respiratory failure [185]. A notable strength of their case report was that they performed a diaphragm biopsy. In a later case report, Rison et al. showed beneficial effects of intravenous immunoglobulin in a patient with DM and bilateral phrenic neuropathy [186].

Brannagan et al. reported a case of a patient with type 1 DM and proximal diabetic neuropathy with decreased respiratory performance [187]. Thus, it is possible that DM may affect multiple respiratory muscles simultaneously and contribute to impaired respiratory performance.

Dharwadkar et al. showed that patients with type 2 DM had lower values of FEV_1_, FVC and maximal expiratory pressure which were independently related to hyperglycemia [188]. These investigators speculated that respiratory muscle dysfunction could underlie the observed effects.

Several studies have pointed towards increased peripheral airway resistance in patients with type 1 DM, which may explain why these patients have a greater breathing effort during exercise [189-191].

In a recently published study, Fuso et al. showed that respiratory muscle strength in patients with type 2 DM was significantly related to metabolic control [192].

Hyperglycemia is implicated in the dysfunction of the diaphragm and other respiratory muscles, which lead to decreased physical performance even in patients without overt cardiopulmonary disease. A summary of the key studies on the impact of DM on respiratory muscle performance is presented in Table 5.

#### DM as a potential risk factor for accelerated decline in lung function

As mentioned above, patients with DM have abnormal DLCO and decreased strength of respiratory muscles. Researchers from Japan and USA showed that patients with DM and no pulmonary disease had lower levels of FVC, which were related to the presence of DM related complications [162,165]. A group from the United Kingdom demonstrated a lower total lung capacity (TLC) in patients with type 1 DM [164]; researchers from Austria showed decreased levels of FEV_1_ and VC, which were associated with the presence of diabetic nephropathy [168].

Lange et al. analyzed data from the Copenhagen City Heart Health Study which included 17,506 adults, 266 of whom were diabetic [193]. They found that patients with DM irrespective of gender had lower values of FEV_1_ and FVC; however, no accelerated decline in pulmonary function was shown during follow up compared with subjects without DM. As discussed above, patients with DM have impaired respiratory muscle performance and decreased physical endurance which may underlie and confound, the decreased pulmonary variables demonstrated in their work. In a similar study, Walter et al. reviewed the data from the Framingham Heart Study [194]. They found that patients with DM had lower levels of FEV_1_ and FVC even after adjustment for smoking, but the diagnosis of DM was not related to COPD independently from gender and smoking.

Lawlor et al. analyzed the data of 3,911 women enrolled in the British Women Heart and Health Study [195]. These researchers showed that the presence of IR and DM were associated with reduced values of FEV_1_. Researchers from Australia studied the data from the Fremantle Diabetes Study and showed that DM was associated with lower values of FEV_1_, VC, FVC and peak expiratory flow (PEF) [196]. More importantly, they found that patients with DM had a greater rate of annual decline in pulmonary function and, in addition, that, DM related airflow limitation was associated with increased mortality.

McKeever et al. analyzed data from the Third National Health and Nutrition Examination Survey to study the association between glucose control and lung function [197]. They showed that patients with DM had lower values of FEV_1_ and FVC, but not a decrease in the ratio of FEV_1_ to FVC. It is pertinent to note that poor control of DM was associated with worse pulmonary function. Litonjua et al. analyzed data from the Normative Aging Study [198]. They demonstrated that patients with DM had lower FEV_1_ and FVC values, even after adjustment for age, gender, smoking, height and weight. However, the presence of DM was not associated with an accelerated decline of pulmonary function in comparison with patients without DM.

Thus, based on the above data, two major possibilities exist: either that, DM independently contributes to the pathophysiology of accelerated decline in pulmonary function or that DM is just a marker for decreased performance of respiratory muscles and physical endurance. A summary of the key studies on the impact of DM on pulmonary function is presented in Table 5.

#### Impact of DM on the COPD outcomes

As mentioned above, the presence of COPD is associated with an increased risk of comorbid diseases and DM in particular [17]. Furthermore, comorbid diseases including DM increase the risk of COPD exacerbation and mortality [16].

As shown by Gan et al. and others, the risk of DM development is associated with elevated fibrinogen and other markers of inflammation [73,199]. In fact, this proinflammatory state may act as an independent risk factor and predictor for COPD exacerbations [200]. Interestingly, a lower DLCO is associated with an increased risk of COPD exacerbation, and as discussed above, DM can be considered as a risk factor for the development of alveolar capilaropathy.

Dahl et al. showed that elevated CRP levels increased the risk of COPD flares [75]. Thus, DM may aggravate the disease course via its proinflammatory profile. It should be emphasized that patients with frequent COPD exacerbation have a much more rapid disease progression and related mortality, which merits particular attention to this clinical group [201].

On the other hand, all types of DM are associated with a significantly increased risk of infections, such as pneumonia and bronchitis [202]. DM associated hyperglycemia may increase the risk of pulmonary infections by making glucose present in the respiratory tree, which in turn predisposes to an infectious complication [203]. On the other hand, airway inflammation can increase local glucose availability, thus causing a vicious cycle. As shown by Phillips et al., glucose in the bronchial tree significantly increased the risk of methicillin resistant staphylococcus aureus (MRSA) infections in mechanically ventilated patients [204]. MRSA infections are well known for difficulties in management and high rates of related morbidity and mortality [205].

McAlister et al. studied the data from 2,471 patients admitted to hospital with community acquired pneumonia (CAP) and analyzed the impact of hyperglycemia on admission to CAP outcomes [206]. These investigators showed that admission glucose levels >11.0 mmol/l were associated with a greater level of in hospital mortality and morbidity. As mentioned previously, researchers from China demonstrated a positive association between IR, hyperglycemia and worsened outcomes during COPD exacerbation [50].

**Figure 3:**
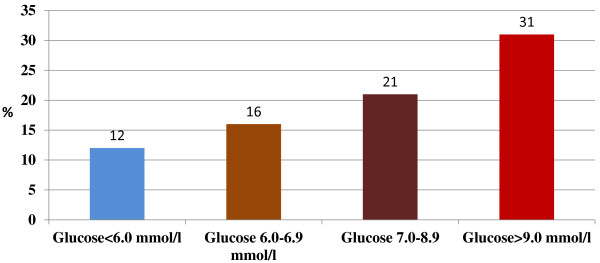
**Serum Glucose, COPD exacerbation and in hospital mortality (adapted from reference # ) **[203]**.**

Baker et al. analyzed the data from 284 patients admitted to hospital with COPD exacerbation [207]. Enrolled patients were subdivided into four groups according to their glucose levels: <6.0 mmol/l, 6.0-6.9 mmol/l, 7.0-8.9 mmol/l and >9.0 mmol/l. This group demonstrated that all levels of increase in glucose were associated with increased COPD related morbidity and mortality. Adapted data from their study is presented in Figure 3.

Chakrabarti et al. studied 88 patients with COPD exacerbation, which required the initiation of non-invasive lung ventilation to assess the impact of glucose control on disease outcomes [208]. These researchers showed that hyperglycemia may be a clinically useful predictor of poor outcomes among patients with severe COPD flare up requiring non-invasive ventilation.

Interesting findings were found by Küpeli et al., who studied 106 patients with COPD, including 29 with MetS to test the impact of MetS on COPD exacerbation [209]. It was shown that patients with MetS had a higher rate of COPD flares, and this was related to an increase in fasting glucose, triglyceride level and CRP. It is plausible that the low grade systemic inflammation seen in MetS contributes to an accelerated progression of COPD. A summary of the key studies on the impact of DM on COPD outcomes is presented in Table 5.

#### DM therapies other than insulin and pulmonary function

As discussed above insulin therapy may improve DLCO, which acts as a surrogate marker of alveolar capillary function. However, inhaled insulin is not generally recommended for widespread use, because of concerns about poor absorption and theoretically plausible side effects.

Kim et al. retrospectively analyzed the data from 61 patients with type 2 DM with a concomitant diagnosis of COPD [210]. These investigators showed that treatment with an oral insulin sensitizer, such as metformin or thiazolidinedione improved FVC in the recruited individuals; this observation was thought to be due to improved respiratory muscle function. However, this study was critically reviewed and potential limitations were highlighted [211]. From a theoretical point of view, antidiabetic medications may improve endothelial function [212], and may also via this mechanism improve the functionality of pulmonary vasculature and gas diffusion.

Moreover, antidiabetic agents have been shown to be associated with a decreased risk of lung cancer in Taiwanese adults [213]. This is particularly relevant, since COPD is considered to be a risk factor for lung cancer independently from smoking [214]. Indeed, metformin can prevent tobacco induced lung carcinogenesis [215] and can activate apoptosis of lung cancer cells [216,217]. As was shown by Tan et al., metformin may improve the efficacy of chemotherapy targeted against non-small lung cancer in patients with type 2 DM [218]. Metformin may be beneficial in the prevention of cancer in general, in patients with type 2 DM and may in fact decrease mortality in patients with cancer and concomitant type 2 DM [219]. However, Bodmer et al. failed to find any effect of metformin administration on the risk of lung cancer [220].

**Figure 4:**
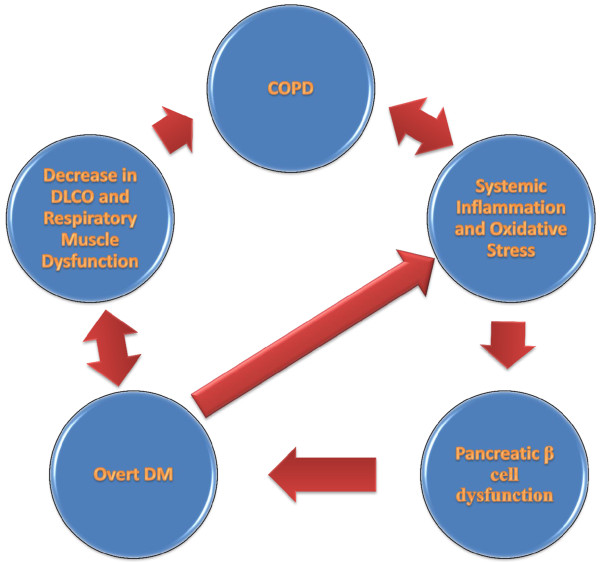
A simplified interrelationship between COPD and DM.

Furthermore, metformin has been shown to ameliorate ventilator induced lung injury, recently demonstrated in a rabbit model [221]. A simplified interrelationship between COPD and DM is presented in Figure 4.

#### OSA, overlap syndrome and the risk of type 2 DM

OSA is a common medical disorder affecting up to 24% of the general adult population [222]. OSA is characterized by repetitive full and/or partial collapses of the upper airway (UA) leading to a state of chronic intermittent hypoxia. OSA is associated with low grade systemic inflammation, oxidative stress and fundamental metabolic abnormalities [223,224], including postprandial dyslipidemia [225], an emerging risk factor for vascular disease [226,227]. These abnormalities are believed to underlie the increased risk for vascular [228-230] and renal disease [231,232] seen in OSA. The disorder is classified based on the number of apnea and/or hypopnea recordings per hour of sleep, which is known as apnea hypopnea index (AHI).

The OSA staging is presented in Table 6. For a more detailed discussion on the topic of general OSA, the reader is referred to a well-written review article [233].

**Table 6:** OSA staging

**OSE Severity**	**Apnea Hypopnea Index**
Mild	5-14
Moderate	15-29
Severe	≥30

The coexistence of OSA and COPD in an individual is called overlap syndrome. Patients with overlap syndrome often have much more advanced cardiopulmonary disease, including pulmonary hypertension, than either disease alone. This is likely explained by far a greater hypoxia, systemic inflammation and oxidative stress from both diseases simultaneously. It is generally thought that it affects approximately 1% of the adult population [234]. However, the epidemiological data are scant.

From a theoretical point, UA collapses in patients with OSA may lead to bronchial obstruction via a reflex mediated pathway [235]. More importantly, patients with overlap syndrome have greater morbidity and mortality. It was shown that continuous positive airway pressure (CPAP) therapy has been shown to reduce the rates of COPD exacerbations [236,237]. For a detailed discussion on the topic of overlap syndrome, the reader is referred to a well-written review article [234].

Unfortunately, the scientific literature on the impact of overlap syndrome on glucose metabolism is scant. However, from a theoretical point of view it is highly likely that it should lead to even greater alteration in glucose metabolism and subsequently to type 2 DM. We will briefly review the current evidence of OSA mediated risk for the new onset type 2 DM.

Several mechanisms are believed to contribute to the pathogenesis of OSA related IR: sleep fragmentation and intermittent hypoxia [238], inflammation and oxidative stress [239,240] and enhanced sympathetic output [224]. It is pertinent to mention that the understanding of how OSA might lead to IR and overt type 2 DM are far from complete. This chapter is not intended to be extensive and the interested reader is referred to some of the well-written review articles on this topic [78,241].

Fredheim et al. showed that OSA is much more common in patients with pre-diabetes and type 2 DM [242]. Indeed, these researchers found that OSA was present in 78% of patients with type 2 DM, compared with 67% of patients with pre-diabetes and 38% of patients without evidence of IR or overt type 2 DM. This was true even after adjustment for age, gender, BMI, insulin sensitivity and high sensitivity for CRP.

Pallayova et al. showed that patients with OSA have lower tissue sensitivity to insulin independent of gender and adiposity [243]. The observed association was believed to be due to an increase in TNF-α and IL-6.

Toqeiro et al. showed that moderate and severe OSA was independently associated with the presence of impaired fasting glucose and markers of IR [244]. Furthermore, several studies showed that OSA is independently related with an increase in glycated hemoglobin [245,246], which is a marker of long term glucose control. Interestingly, it was recently shown that even lean subjects with OSA have greater insulin levels as a result of IR [247], which supports an independent role of OSA in the development of type 2 DM.

Several prospective studies showed that OSA is an independent risk factor for the development for type 2 DM [248,249] and a recently published meta-analysis of prospective studies confirmed this relationship [250].

Sharma et al. studied 86 patients with OSA to assess the impact of CPAP treatment on the metabolic profile, including the IR measurement and glycated hemoglobin in a double-blind placebo (sham CPAP)-controlled trial [251]. These researchers showed that CPAP therapy was significantly associated with a statistically significant reduction in glycated hemoglobin levels. Weinstock et al. showed that CPAP treatment of severe OSA is associated with an improvement in insulin sensitivity [252]. It also has been recently shown that CPAP treatment is independently related to an increase in peripheral insulin sensitivity in patients with concomitant acromegaly [253]. This is particularly important since acromegaly is associated with both type 2 DM and OS [254]. A recent meta-analysis performed by Yang et al. supported the beneficial effects of CPAP on the glucose metabolism [255].

In concluding this section it is necessary to note that emerging evidence suggests that OSA might be an independent risk factor for the development of non-alcoholic fatty liver disease [256], which is associated with IR and overt type 2 DM [257], as well as vascular [258] and renal diseases [259].

**Figure 5:**
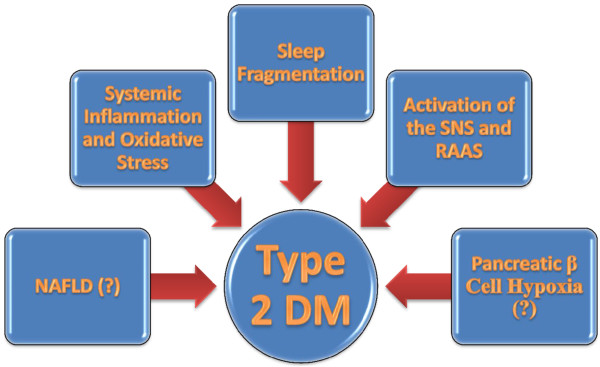
Pathophysiology of OSA mediated type 2 DM.

Based on the current evidence, OSA should be considered as an independent risk factor for the development of type 2 DM, and, when coexisting with COPD, the risks are likely to be higher. However, it is essential to note that scientific literature is scant on the topic of overlap syndrome and the risks of type 2 DM. A simplified sketch on how OSA may increase the risk of type 2 DM is shown in Figure 5.

## Conclusion

COPD, MetS and DM are common and underdiagnosed medical conditions. It is predicted that COPD will be the third leading cause of death worldwide by 2020. The burden of this disease is even greater if we consider the significant impact of COPD on cardiovascular mortality.

COPD may be considered as a novel risk factor for new onset type 2 DM. The pathophysiology of this is likely to be very complex with several factors being involved, including: inflammation and oxidative stress, administration of glucocorticosteroids, COPD related skeletal muscle dysfunction and abnormalities in adipokine metabolism etc. However, COPD should not be considered as a risk factor for type 1 DM, because of the unique pathophysiology of type 1 DM and different ages at disease presentation.

On the other hand, diabetes may act as an independent factor negatively affecting lung structure and function. Diabetes can cause muscle and neuronal damage, which is relevant to deficient function of respiratory muscles. Moreover, diabetes is independently associated with lower physical performance, which can be disabling for patients with COPD, who already have some limitation in physical performance. DM is able to detrimentally affect alveolar capillary membrane and decrease DLCO, similarly to other microangiopathic complications, such as diabetic nephropathy. Furthermore, DM is associated with the presence of glucose in airway secretions, and this may contribute to the increased risk of pulmonary infections seen in diabetics. MetS can increase the risk of COPD exacerbation, and diabetes is associated with worsened outcomes of COPD flares. On the top of that, coexistent OSA may increase the risk for type 2 DM in some individuals.

Antihyperglycemic medications may in fact improve DLCO, as has been shown with insulin in patients with DM. However, concerns about safety and pharmacokinetics preclude the recommendation for inhaled insulin to be used at this time. On the other hand, oral antihyperglycemic medications such as metformin and thiazolidinedione may improve FVC in patients with DM. Moreover, metformin has been shown to have antitumor effects and may increase survival in patients with lung cancer.

Thus, it is essential to look at COPD as a potential independent risk factor for the incidence of MetS and type 2 DM and for a complicated course of DM. Conversely, both types of DM and MetS are associated with a worsened clinical course of COPD and a greater degree of morbidity and mortality.

## Abbreviations

AHI: Apnea hypopnea index; BA: Bronchial asthma; BMI: Body mass index; CAP: Community acquired pneumonial; CI: Confidence interval; COPD: Chronic obstructive pulmonary disease; CPAP: Continuous positive pressure therapy; CRP: C-Reactive Protein; DLCO: Diffusing capacity for CO; DM: Diabetes Mellitus; ERV: Expiratory reserve volume; FEV_1_: Forced expiratory flow in 1 second; FRC: Functional residual capacity; FVC: Forced vital capacity; GLUT: Glucose transporter; GOLD: Global initiative for chronic Obstructive lung disease; HF: Heart failure; HIF: Hypoxia inducible factor; HOMA: Homeostatic model assessment; HR: Hazard ratio; IL: Interleukin; IR: Insulin resistance; MetS: Metabolic syndrome; MMEF: Maximum midexpiratory flow; MRSA: Methicillin resistant staphylococcus aureus; NAFLD: Non alcoholic fatty liver disease; NHANES: National health and nutrition examination survey; NIV: Non-invasive ventilation; OSA: Obstructive sleep apnea; OR: Odds ratio; PEF: Peak expiratory flow; RAAS: Renin angiotensin aldosterone system; RR: Relative risk; RV: Residual volume; SNP: Single nucleotide polymorphism; SNS: Sympathetic nervous system; TLC: Total lung capacity; TNF-α: Tumor necrosis factor alpha; UA: Upper airway; VA: Alveolar ventilation; VC: Vital capacity.

## Competing interest

The author has no competing interests.
